# Adult-onset Still’s disease with disseminated intravascular coagulation and hemophagocytic syndrome: a case report

**DOI:** 10.1186/1756-0500-7-940

**Published:** 2014-12-22

**Authors:** Tetsuhiko Mimura, Masanori Shimodaira, Minoru Kibata, Akihiro Tsukadaira, Kumiko Shirahata

**Affiliations:** Iida Municipal Hospital, Nagano, Japan; Department of Internal Medicine, Iida Municipal Hospital, 438 Yawata-machi, Iida, Nagano, 395-8502 Japan

**Keywords:** Still’s disease, Disseminated intravascular coagulation, Hemophagocytic syndrome

## Abstract

**Background:**

Adult-onset Still’s disease is a rare inflammatory condition of unknown origin characterized by high spiking fever, arthralgia, arthritis, myalgia, salmon-colored evanescent rash, and hepatosplenomegaly. The diagnosis of adult-onset Still’s disease requires the exclusion of other possible disorders because it lacks specific clinical and histopathological findings. Adult-onset Still’s disease rarely become fatal due to visceral involvements such as disseminated intravascular coagulation.

**Case presentation:**

A 22-year-old Chinese female presented to our medical center with high spiking fever for one week, myalgia for two weeks, and arthralgia and pink maculopapular rash for four weeks. She developed disseminated intravascular coagulation on the fourth day after admission. There was no other explanation for the fever and rash, including infection, malignancy, and collagenosis. Together, the high spiking fever, salmon-colored rash, splenomegaly, and excess hepatic enzyme, indicated adult-onset Still’s disease based on the Yamaguchi criteria. Therefore, prednisolone therapy was initiated. The combination of nafamostat mesilate and prednisolone therapies caused a rapid reduction in the fever and rash. The inflammatory markers decreased immediately, and disseminated intravascular coagulation improved. Her symptoms resolved with low-dose prednisolone treatment, and she was monitored thereafter at our outpatient clinic.

**Conclusion:**

The previous use of nonsteroidal anti-inflammatory drugs could have caused disseminated intravascular coagulation in this patient with adult-onset Still’s disease. We propose that physicians should consider the possibility of disseminated intravascular coagulation as a complication during the course of adult-onset Still’s disease and suggest that prednisolone therapy should be initiated in the early stages of adult-onset Still’s disease.

## Background

Adult-onset Still’s disease (AOSD) is a systemic inflammatory disorder of unknown etiology characterized by high spiking fever, arthralgia, arthritis, myalgia, salmon-colored evanescent rash, and hepatosplenomegaly [1, 2]. The diagnosis of AOSD requires the exclusion of other possible disorders because it lacks specific clinical and histopathological findings [3, 4]. During the course of AOSD, life-threatening conditions such as hepatic involvement, cardiac tamponade, respiratory distress syndrome, or pancytopenia caused by hemophagocytic syndrome (HS) occasionally develop [5]. However, cases of AOSD with disseminated intravascular coagulation (DIC) are not common [6–10]. We report a case of AOSD with DIC, which was dramatically improved by prednisolone.

## Case presentation

**Figure 1:**
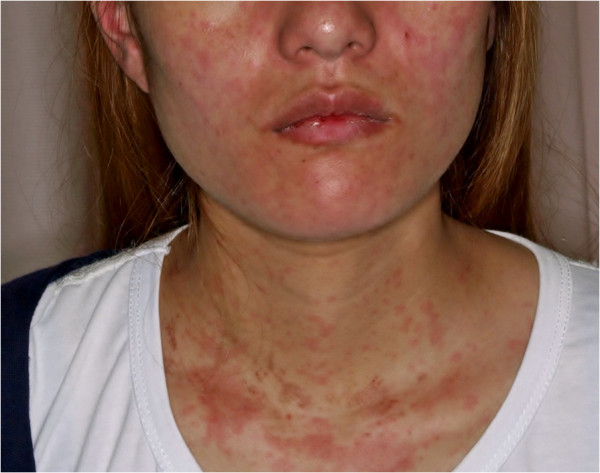
**Picture of the patient showing salmon-colored rash on her face and trunk.**

A 22-year-old Chinese female presented to our medical center with a high spiking fever and a pink maculopapular rash on the trunk, face, and limb for one week (Figure 1), myalgia for two weeks, and arthralgia for four weeks. Her fever was between 35°C and 39°C; its occurrence correlates with the appearance of the rash, and it was not relieved by treatment. She had been taking NSAID called loxoprofen for her fever for a few days, without improvement. The patient did not have allergies, a past medical history, alcoholism, herbal treatment, insect bites, or contact with any animal. She did not travel to any foreign country for the past two years.

Physical examination revealed high fever, a regular pulse of 100 beats per min, and normal blood pressure (116/84 mmHg). There were no signs of anemia, jaundice, lymph node swelling, hepatosplenomegaly or goiter. Auscultation of the lungs revealed no rale, and her heart function sounded normal. Myalgia was detected in the arms and legs by pressure algometry.

Blood sample analysis revealed high levels of C-reactive protein (3.03 mg/dL; normal: 0–0.3 mg/dL), lactate dehydrogenase (751 IU/L; normal: 109–244 IU/L), aspartate transaminase (76 IU/L; normal: 10–40 IU/L), ferritin (1027 ng/ml; normal: 10–291 ng/ml), and creatinine phosphokinase (239 IU/L; normal: 40–149 IU/L). In contrast, the patient had normal white blood cell counts (6.4 × 10^3^/μL; normal: 3.1-8.0 × 10^3^/μL), hemoglobin (12.2 g/dL; normal: 10.1-14.5 g/dL), and platelet count (10.5 × 10^4^/μL; normal: 11.0-34.0 × 10^4^/μL), γ-glutamyltransferase (18 IU/L; normal: 8–68 IU/L), alanine transaminase (24 IU/L; normal: 5–44 IU/L) and alkaline phosphatase (173 IU/L; normal: 80–260 IU/L).

Serology tests were negative for the rheumatoid factor, anti-nuclear antibodies, anti-DNA antibodies, anti-neutrophil cytoplasmic antibodies, and anti-Jo-1 antibodies. There was no marker of recent infection, including hepatitis B antigen, hepatitis C virus, HIV antibodies, Mycobacterium tuberculosis antigen, Epstein–Barr virus, cytomegalovirus, herpes simplex viruses, mycoplasma pneumonia, Human parvovirus B19, Rickettsia japonica, or Orientia tsutsugamushi.

**Figure 2:**
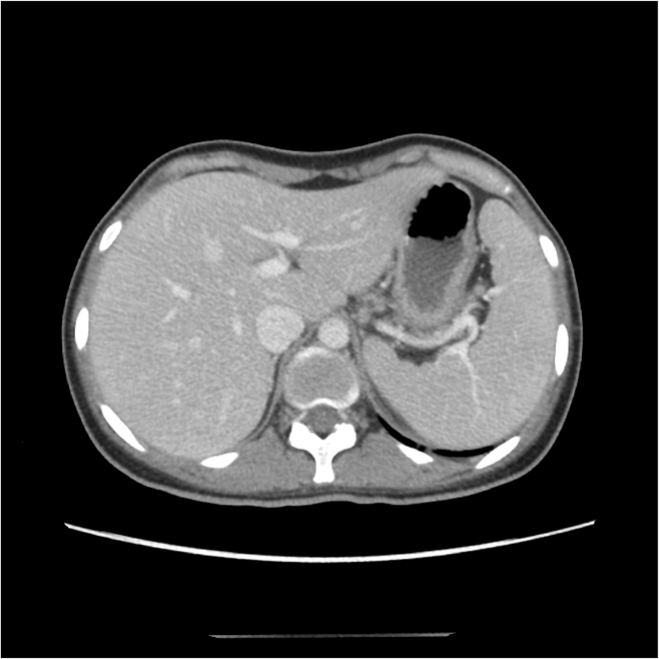
**Computed tomography images showing splenomegaly.**

Computed tomography (CT) images revealed splenomegaly, in the absence of abscess or tumor (Figure 2). Echocardiography was negative for endocarditis.

**Figure 3:**
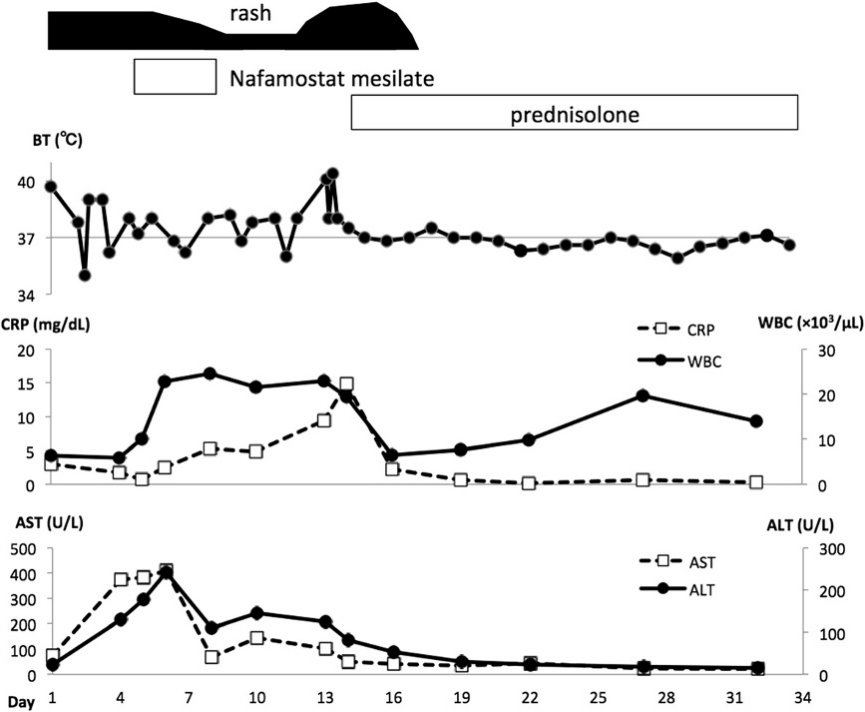
**Clinical course of a patient with adult-onset Still’s disease.** ALT, alanine transaminase; AST, aspartate transaminase; BT, body temperature; CRP, C-reactive protein; WBC, white blood cell count.

After admission, the high fever persisted, with increasing levels of hepatic and biliary enzymes (Figure 3). On day 4 after admission, the platelet count suddenly decreased to 6.3 × 10^4^/μL, and the fibrinogen level decreased to 122 mg/dL (normal: 190–430 mg/dL). In contrast, the patient had excess fibrin degradation product (FDP; 45.5 μg/mL; normal: 1–10 μg/mL), and a high prothrombin time-international normalized ratio (PT-INR: 1.23; control: 1.0). Based on these data, she was diagnosed with DIC and nafamostat mesilate (200 mg/day) therapy was initiated immediately.

**Figure 4:**
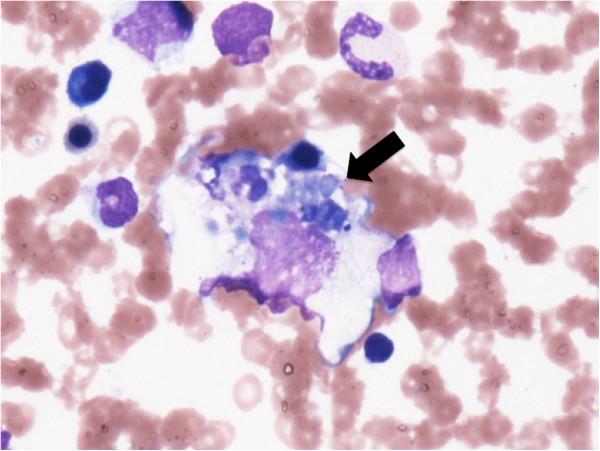
**Bone marrow showing hemophagocytosis (black arrow).**

A bone marrow biopsy showed hemophagocytosis, no blast cells, and no malformation (Figure 4). Because the patient had fever, splenomegaly, and hypofibrinogenemia, but no evidence of malignancy, we diagnosed HS on the basis of these findings [11].

There was no other explanation for the fever and rash, including infection, malignancy, and collagenosis. Together, the high spiking fever, salmon-colored rash, splenomegaly, and excess hepatic enzyme, indicated AOSD based on the Yamaguchi criteria [9]. Therefore, prednisolone (30 mg/day) therapy was initiated.

The combination of nafamostat mesilate and prednisolone therapies caused immediate improvement of both the fever and rash. The inflammatory markers decreased immediately, and DIC improved. On day 34, the dosage of oral prednisolone was decreased from 30 mg/day to 25 mg/day, and the patient was discharged. Her symptoms resolved upon treatment with low-dose prednisolone, and she was monitored thereafter at our outpatient clinic.

## Conclusions

The present case highlights the difficulty of diagnosing AOSD. Moreover, our case showed that AOSD could be a cause of DIC. The diagnosis of AOSD is clinically important and difficult because the disorder has no specific laboratory marker. The Yamaguchi criteria are widely recognized as the most sensitive tool to diagnose AOSD [12]. Nonetheless, differential diagnosis is necessary to exclude other diseases, such as an infection (i.e., endocarditis), neoplastic diseases (i.e., lymphomas), or autoimmune diseases (i.e., vasculitis and polymyositis) [13, 14]. In this case, infection, neoplastic disease, or autoimmune disease was not revealed by an extensive examination that included blood test, CT scan, and bone marrow biopsy.

In general, the treatment of AOSD involves corticosteroids, generally medium-high to high doses (0.5–1 mg/kg/day) of prednisone equivalent [14]. For AOSD patients with severe visceral involvement, intravenous infusion of high-dose methylprednisolone generally provides efficient symptom relief. The corticosteroid response should be detected within a few hours or days [14]. In our case, prednisolone therapy dramatically reduced the fever and rash within 3 days.

In patients with AOSD, macrophage hyperactivation and cytokine overproduction cause liver damage [14, 15]. The onset of DIC is precipitated by a decrease in procoagulant factor caused by liver dysfunction [16]. In some reports, the development of DIC in patients with AOSD was attributed to the use of nonsteroidal anti-inflammatory drugs (NSAIDs) [17, 18]. It is surmised that the disease-induced hepatic damage, further aggravated by aspirin, may be a significant factor in the initiation and perpetuation of intravascular coagulation [17]. Accordingly, it is critical to monitor liver enzymes during the initial stage of AOSD therapy because NSAIDs may induce severe hepatitis [3]. Another study suggests that high serum levels of unbound anti-inflammatory drugs, due to reduced serum albumin binding, may cause intravascular coagulation by their effect on prostaglandin synthesis [18]. Because our patient was taking loxoprofen before her admission to our hospital, this NSAID may have triggered DIC.

Bone marrow analysis revealed that our patient developed from HS. This condition is caused by hyperactivated T cells (Th1 cells) and macrophages, and the subsequent overproduction of cytokines such as IL-1, IL-6, and IFN-γ [19]. These cytokines contribute to the development of thrombotic reactions by activation of vascular endothelial cells and monocytes [20]. Earlier cases of AOSD complicated with DIC and HS were treated successfully with NSAIDs, steroids, nafamostat mesilate, gabexate mesilate, or cyclosporine [6, 7, 21]. To avoid the use of NSAIDS, our patient was treated with nafamostat mesilate and steroid therapy. She responded well to the treatment and was discharged on low-dose prednisolone.

In conclusion, we report a case of AOSD complicated by HS and then DIC. The onset of DIC could be due to the use of NSAIDs. The differential diagnosis of AOSD requires several tests to rule out infections, neoplastic diseases, and autoimmune diseases. However, patients with AOSD may rapidly develop life-threatening conditions such as DIC. Therefore, we propose that physicians should be alert to the possibility of DIC as a complication during the course of AOSD and that prednisolone treatment should be initiated in the early stages of AOSD.

## Consent

Written informed consent was obtained from the patient for publication of this Case Report and any accompanying images. A copy of the written consent is available for review by the Editor-in-Chief of this journal.

## Abbreviations

AOSD: Adult-onset Still’s disease
DIC: Disseminated intravascular coagulation
CT: Computed tomography
HS: Hemophagocytic syndrome
FDP: Fibrin degradation product
PT-INR: Prothrombin time-international normalized ratio.
